# Patterns and profiles of drug resistance-conferring mutations in *Mycobacterium tuberculosis* genotypes isolated from tuberculosis-suspected attendees of spiritual holy water sites in Northwest Ethiopia

**DOI:** 10.3389/fpubh.2024.1356826

**Published:** 2024-03-19

**Authors:** Melese Abate Reta, Nontuthuko Excellent Maningi, P. Bernard Fourie

**Affiliations:** ^1^Department of Medical Microbiology, Faculty of Health Sciences, University of Pretoria, Pretoria, South Africa; ^2^Department of Medical Laboratory Science, College of Health Sciences, Woldia University, Woldia, Ethiopia; ^3^Department of Microbiology, School of Life Sciences, College of Agriculture, Engineering and Science, University of KwaZulu-Natal, Durban, South Africa

**Keywords:** drug-resistant TB, rpoB, katG, gyrA, mutations, spoligotypes, pulmonary tuberculosis, spiritual holy water site attendees

## Abstract

**Purpose:**

This study examined the patterns and frequency of genetic changes responsible for resistance to first-line (rifampicin and isoniazid), fluoroquinolones, and second-line injectable drugs in drug-resistant *Mycobacterium tuberculosis* (MTB) isolated from culture-positive pulmonary tuberculosis (PTB) symptomatic attendees of spiritual holy water sites (HWSs) in the Amhara region.

**Patients and methods:**

From June 2019 to March 2020, a cross-sectional study was carried out. A total of 122 culture-positive MTB isolates from PTB-suspected attendees of HWSs in the Amhara region were evaluated for their drug resistance profiles, and characterized gene mutations conferring resistance to rifampicin (RIF), isoniazid (INH), fluoroquinolones (FLQs), and second-line injectable drugs (SLIDs) using GenoType®MTBDR*plus* VER2.0 and GenoType®MTBDR*sl* VER2.0. Drug-resistant MTB isolates were Spoligotyped following the manufacturer’s protocol.

**Results:**

Genetic changes (mutations) responsible for resistance to RIF, INH, and FLQs were identified in 15/122 (12.3%), 20/122 (16.4%), and 5/20 (25%) of MTB isolates, respectively. In RIF-resistant, *rpo*B/Ser**531**Lue (*n* = 12, 80%) was most frequent followed by His**526**Tyr (6.7%). Amongst INH-resistant isolates, *kat*G/Ser**315**Thr1 (*n* = 19, 95%) was the most frequent. Of 15 MDR-TB, the majority (*n* = 12, 80%) isolates had mutations at both *rpo*B/Ser**531**Leu and *kat*G/Ser**315**Thr1. All 20 INH and/or RIF-resistant isolates were tested with the MTBDR*sl* VER 2.0, yielding 5 FLQs-resistant isolates with gene mutations at *rpo*B/Ser**531**Lue, *kat*G/Ser**315**Thr1, and *gyr*A/Asp**94**Ala genes. Of 20 Spoligotyped drug-resistant MTB isolates, the majority (*n* = 11, 55%) and 6 (30%) were SIT149/T3-ETH and SIT21/CAS1-Kili sublineages, respectively; and they were any INH-resistant (mono-hetero/multi-). Of 15 RIF-resistant (RR/MDR-TB) isolates, 7 were SIT149/T3-ETH, while 6 were SIT21/CAS1-Kili sublineages. FLQ resistance was detected in four SIT21/CAS1-Kili lineages.

**Conclusion:**

In the current study, the most common gene mutations responsible for resistance to INH, RIF, and FLQs were identified. SIT149/T3-ETH and SIT21/CAS1-Kili constitute the majority of drug-resistant TB (DR-TB) isolates. To further understand the complete spectrum of genetic changes/mutations and related genotypes, a sequencing technology is warranted.

## Background

Drug-resistant *Mycobacterium tuberculosis* (MTB) is an escalating health problem that presents substantial challenges for public health systems across the world (1–3). The advent and transmission of drug-resistant MTB strains, especially multidrug-resistant/RIF-resistant TB (MDR/RR-TB)-which is resistant to the two key anti-mycobacterial drugs, INH and RIF- and “extensively drug-resistant TB (XDR-TB)”-which is an MDR/RR isolate that is also resistant to any FLQs and bedaquiline and/or linezolid-has made it more challenging to contain and eradicate the disease globally (1–4). It makes TB control programs less effective since resistant TB strains require specialized laboratory facilities, diagnostic tools with higher accuracy, and treatment regimens that aren’t usually available in resource-constrained settings (1, 2). Globally, 10.6 million people contracted TB, and about “410,000 developed MDR or RIF-resistant TB” (5). The prevalence of DR-TB varies by geography, with India, China, and the Russian Federation having the largest burden (1). Ethiopia bears a substantial burden of TB, and the rising incidence of DR-TB has detrimental effects on the country’s health systems and efforts to contain the disease (1, 6–8). Moreover, although Ethiopia is on track to transition out of the thirty nations with high rates of MDR/RR-TB (1), the burden of resistant TB is still a problem, where 0.71% of new TB cases and 12.0% of retreated TB patients were reported to have MDR-TB (9).

Drug-resistant TB strain has a severe impact on both individual and societal levels, with resistant TB being associated with higher patient mortality rates than susceptible TB strains (1, 3, 10). The treatment outcomes for DR-TB are often unfavorable due to the limited accessibility and effectiveness of second-line anti-TB medicines, lengthy treatment durations, and higher toxicity of the drugs (2, 10). Thus, patients experience prolonged suffering and there is an increasing risk of transmitting the disease to others (3, 11). Accordingly, DR-TB necessitates an accurate and timely diagnosis for the effective management of TB patients (3).

Molecular diagnostic methods have transformed the detection and identification of drug-resistance-associated genetic changes in MTB in a rapid and precise manner (2, 12), providing accurate evidence for patient-centered treatment decisions (13). The application of “PCR-based tools, like the GeneXpert®MTB/RIF (Cepheid Inc., Sunnyvale, CA, USA),” has significantly transformed the process of detecting TB and drug-resistant TB and enhanced the treatment of TB patients (14). “Line Probe Assays (LPAs) (Hain Lifescience, Germany)” are another widely used tool for the simultaneous diagnosis of TB and the anti-mycobacterial resistance profile of TB strains (14, 15). These assays target specific genetic regions linked with resistance to INH and RIF, and FLQs and SLIDs, respectively. Although LPAs have certain limitations that only detect the gene mutations within the target of interest (15), “MTBDR*plus* VER 2.0” (16), and “MTBDR*sl* VER 2.0” (17), have higher sensitivity and specificity than the first version to diagnose anti-TB drug resistance, and associated gene mutations (16, 17). Ethiopia is one of the nations that has benefited from the use of LPA diagnostic technologies to improve the management of TB patients. LPAs (“MTBDR*plus* and MTBDR*sl*”) tests were integrated into the Ethiopian national TB diagnostic algorithm (18, 19). Since the rollout of LPAs in Ethiopia, TB diagnostic referral centers at the national and regional levels have been authorized to undertake those assays (19).

Genetic mutations play a crucial role in conferring anti-TB drug resistance in MTB (2). The presence of those genetic changes conferring resistance to key anti-mycobacterial drugs differs geographically and is influenced by factors like the prevalence of DR-TB strains, treatment regimens, and patient adherence to therapy (20). Thus, understanding the types and frequency of those genetic changes is critical for effective drug-resistant strain detection, guiding treatment strategies, and monitoring the emergence of new resistance patterns (20, 21).

Furthermore, it is paramount to identify populations at high risk for TB transmission and conduct TB case finding in hotspot settings (22, 23). The Ethiopian National TB Control Program identified prisoners, HIV-positive people, diabetic patients, the older adult, University residents, TB patients’ contacts, the homeless, healthcare workers, and refugees as key high-risk groups for TB (22); thereby earlier studies reported the burden of TB in prison settings (24, 25), refugees (26), homeless (27, 28), and University residents (29), in Ethiopia. Although Ethiopia has identified key populations for TB and implemented WHO’s policy on TB infection control in high-risk settings, targeting spiritual holy water sites as high-priority settings for TB control activities is not given much attention (30). The first national TB prevalence survey (6), and an earlier community-based TB study among key populations in hotspot settings in Ethiopia have been conducted (31). However, these studies excluded high-risk places such as holy water sites and other congregate settings, which can be potential hotspots for TB transmission. The current study was guided or aimed to answer the question, “What is the prevalence of TB, drug-resistant rate, the genotype distribution of resistant MTB isolates, and the frequency of gene mutations responsible for resistance for first and second-line anti-TB drugs among PTB suspects attending holy water site in the Amhara region?” As a baseline information, an earlier study on the prevalence of smear-positive TB among holy water site attendees was conducted in the Amhara region and found that the prevalence of TB was 7.4-fold higher in those study cohorts than in the general population in Ethiopia (32). However, this study was geographically limited and used acid-fast bacilli (AFB) smear microscopy to detect TB suspects, which has low sensitivity, thus the findings may not reflect the true TB burden among holy water site attendees in the region (32).

Spiritual holy water sites in Ethiopia are congregate settings where many people, particularly Orthodox Christian followers, come to receive holy water treatment blessed by priests for several kinds of illnesses, including TB and other respiratory diseases (32). Although studies showed that TB patients seek treatment from holy water sites, the prevalence of TB among individuals attending those settings in Ethiopia has not been thoroughly investigated. There is also scarce information on the genetic diversity of drug-resistant MTB genotypes and the types and frequency of genetic changes conferring resistance to commonly prescribed anti-TB drugs among TB isolates from PTB-symptomatic attendees of holy water sites in Ethiopia. Therefore, this study aimed to evaluate drug susceptibility profiles of TB isolates and characterize the types and frequency of mutations responsible for resistance to INH, RIF, FLQs, and SLIDs in MTB isolates, and to Spoligotype those isolates from culture-positive PTB-symptomatic attendees of holy water sites in the Amhara region, Ethiopia.

## Methods

### Setting and study period

A cross-sectional study was performed from June 2019 to March 2020 at nine purposively selected holy water sites found across nine administrative zones in the Amhara region. The Amhara region is found in Northwestern Ethiopia, which comprises thirteen zones and three administrative towns. Bahir Dar is the regional capital, which is 567 km far from the national capital, Addis Ababa. One holy water site was chosen from each administrative zone based on its consistent popularity for spiritual holy water treatment, its ability to accommodate a large number of attendees, and where many people visit and reside for an extended time (32). According to the criteria mentioned above, the selected holy water site from each study zone is deemed to be representative (Supplementary Table S1).

### Study participants

For this study, the source population was all individuals who attended holy water sites during the data collection time. However, attendees who had PTB suggestive symptoms (33), particularly cough ≥2 weeks and having productive cough, and other symptoms such as night sweating, fatigue, fever, loss of appetite, shortness of breath, chest pain, unexplained weight loss, had contact history with active TB patients, and previous TB disease were recruited and included as the sample population. During the course of the study, at nine selected holy water sites, a total of 10,313 attendees (≥18 years of age) were screened, and 560 individuals with symptoms of PTB participated. Supplementary Table S1 illustrates the study settings, total number of attendees screened for PTB-suggestive symptoms, number of PTB suspected cases, and bacteriologically confirmed TB cases.

### Eligibility criteria

Attendees (≥18 years of age) who had coughs ≥2 weeks plus the above-mentioned PTB suggestive symptoms were included, whereas individuals who were under 18 years and those who were seriously ill to provided sputum sample and necessary information were excluded. Furthermore, attendees who were on TB medications during the sample collection time were excluded.

### Sociodemographic data collection

After obtaining participants’ informed consent to be included in the study, their sociodemographic data including sex, age, residence, marital status, educational level, family size per household, occupation, and study area were recorded using an interviewer-administered questionnaire. We followed the national TB screening guideline during the screening of PTB suggestive symptoms (33).

### Sputum specimen collection and *Mycobacterium tuberculosis* complex isolates

Using a leak-proof, sterile screw-capped falcon tube (50 mL capacity), the sputum samples were received from PTB-symptomatic attendees. We used an ice-pack carrier to transport the sputum specimens to the regional research laboratory center, “Amhara Public Health Institute, Bahir-Dar, Ethiopia.” We followed the standard procedures during the mycobacterial culturing steps. In brief, the “N-acetyl-L-cysteine (NALC-NaOH)” solution was utilized to decontaminate the sputum, following the neutralization process using a phosphate buffer solution (pH 6.8). The mixture was centrifuged to prepare the inoculums for culture. Then after, the inoculum/sediment from the bottom of the tube was taken and inoculated into Löwenstein-Jensen (LJ) slant culture tubes, and incubated at a temperature of 37°C for at least 8 weeks. The growth of MTB colonies on LJ culture media was inspected weekly for up to 8 weeks, and the growth was confirmed by Ziehl-Neelsen (ZN) smear staining” (34). “Capilia TB-Neo (Tauns laboratories, Japan)” was utilized to differentiate MTB complex species (35). In each step of the test, a known H37Rv strain and sterile molecular-grade water were utilized as positive and negative controls, respectively.

### Specimen preparation from MTB colonies grown on LJ culture media

From MTB colonies grown from LJ culture medium, suspensions were prepared and transferred into a transport media, 1.5 mL of “PrimeStore Molecular Transport Medium (PS-MTM; Longhorn Vaccine & Diagnostics, San Antonio, TX, USA).” The MTB suspension preparations from a positive LJ culture medium depend on the culture state (36). In summary, for intact slopes, using a sterile inoculation loop, MTB colonies were carefully scraped off and washed down (suspended) in 1 mL sterile molecular grading water in the original slant culture bottle. After pipetting off the prepared suspension, it was transferred into a 1.5 mL Eppendorf tube. The Eppendorf tubes holding suspensions were centrifuged at 13,000 g for 5 min, the supernatant was discarded (36), and the deposit was then transferred into the PS-MTM. The PrimeStore tubes holding the MTB suspension were shipped non-refrigerated to South Africa by air, where DNA extraction, genotyping drug susceptibility testing (gDST), and Spoligotyping procedures were performed.

### *Mycobacterium tuberculosis* genomic DNA extraction

From all LJ culture-positive isolates, the “MTB genomic DNA was extracted using the PrimeXtract™ kit (Longhorn Vaccines and Diagnostics, San Antonio, TX, USA)” following the manufacturer’s protocol. In summary, MTB inoculum (200 μL), 100% ethanol (200 μL), and lysis buffer (200 μL) were transferred into a 1.5 mL microcentrifuge tube, then thorough vortexing and subsequent centrifugation was performed. Using a micro-extraction column, the entire supernatant was transferred, following centrifugation at 13,000 rpm for 1 min, and then the follow-through material was discarded. After adding wash buffer 1 (500 μL) to the extraction column, it was then centrifuged at 13,000 rpm for 1 min, followed by further addition of wash buffer 2 (500 μL) to the extraction columns, and subsequently centrifuged as described above, and the follow-through material was discarded. The extracted total MTB genomic DNA was eluted by centrifugation at 13,000 rpm for 1 min using preheated (60–70°C) 50 μL elution solution. Then, the extracted total DNA was stored at −20°C fridge for future use. Genomic MTB DNA concentration and quality were measured using a spectrophotometer at optimal densities of 269 and 280 nm.

### Drug susceptibility testing

The genotype DST (gDST), MTBDR*plus* VER2.0 (“Hain Lifescience, Germany”) was performed on 122 culture-positive MTB isolates (16). MTB isolates that were found RIF and/or INH-resistant were subjected to MTBDR*sl* VER2.0 to detect FLQs and second-line injectable anti-mycobacterial drug resistance (17). The entire techniques that included the preparation of the master mix, polymerase chain reaction (PCR) amplification, and hybridization were done following the manufacturer’s protocol (“Hain Lifescience, Germany”) (16, 17).

### Interpretation of results

The MTBDR*plus* assay can detect the presence and absence of wild-type (WT) and mutant (MUT) DNA sequences (bands) within particular regions of the three resistance-conferring genes: the *rpo*B gene, which encodes the “*β*-subunit of the RNA polymerase,” was used to determine RIF-resistance; whereas the *kat*G gene, which encodes the “catalase-peroxidase,” was used to detect high-level INH resistance; and the *inh*A promoter region (encodes enoyl ACP NADH reductase), was used to identify low-level INH resistance. Rifampicin resistance was demonstrated by the absence of bands in the *rpo*B probes, whereas resistance to INH was demonstrated by the absence of *kat*G and *inh*A bands. On the other hand, when the mutant probes’ bands are as strong as or stronger than the existing amplification control (AC) bands, they are considered resistant. In most cases, the absence of the WT band is associated with the presence of a MUT band, indicating resistance. Rarely, the absence of WT band (s) without the presence of a corresponding MUT band could be observed, which was attributed to “unknown” genetic changes in the probe region or mutations that exist outside of the drug-resistance-determining regions, which the assay cannot detect. The coexistence of WT and MUT bands within a single stripe may indicate the existence of hetero-resistance or mixed TB strain infection. In general, resistance was recorded where one or more WT bands were absent or where one or more WTs were missing with a corresponding mutation.

The MTBDR*sl* VER2.0 test determined the presence and absence of WT and MUT DNA sequences within a particular region of four genes: *gyr*A, which encodes the “A-subunit protein of DNA gyrase; *gyr*B (it encodes *β* -subunit protein of DNA gyrase)” were utilized to determine resistance to FLQs; and the *rrs* (encodes 16S rRNA) was utilized for the detection of cross-resistance to Kanamycin (KAM) and Amikacin (AMK), and Capreomycin (CPM) and Viomycin (VIO), whereas the *eis*, which encodes (aminoglycoside acetyltransferase), was used to identify low-level resistance to KAM. In short, resistance was recorded where one or more WT probes were absent or where one or more WTs were missing with a corresponding mutation type. However, the probability of strains developing mutations outside of the test regions cannot be ruled out, since the assay cannot detect genetic changes that exist outside the regions of interest.

### Quality control

In each of the LPAs tests (MTBDR*plus* VER2.0 and MTBDR*sl* VER2.0), sterile water was utilized as the negative control, while the universal reference H37Rv strain, which is sensitive to all anti-TB medicines, was utilized as a positive control.

### Spoligotyping

All 20 drug-resistant MTB isolates were subjected to Spoligotyping “following the manufacturer’s protocol” (37), and the Spoligotyping kit supplier’s instructions (“Ocimum Biosolutions Company, IJsselstein, The Netherlands”). In brief, the MTB isolate’s “direct repeat (DR) region was amplified by a thermal cycler PCR machine” (“VWR International, UK”) utilizing the oligonucleotide primers derived from the MTB direct repeat region. A total volume of 25 μL constituting the PCR amplification reaction mixture of 12.5 μL of “HotStarTaq Master Mix (Qiagen, UK),” 2 μL of forward primer (DRa), and 2 μL of reverse primer (DRb), 5 μL of extracted DNA, and 3.5 μL sterile molecular grading water. Then, the mixture was subjected to heat at 96°C for 3 min, and then subjected to 30 cycles for 1 min at 96°C, 1 min at 55°C, 30 s at 72°C, and 5 min at 72°C for 1 cycle.

The amplified PCR product underwent hybridization with a series of 43 immobilized oligonucleotides that are covalently bound to a membrane (“Animal and Plant Health Agency, Great Britain”). Each of these oligonucleotides corresponds to a different spacer sequence of DNA located within the direct repeat (DR) locus. The membrane was washed twice for 10 min in 2XSSPE (pH 7.7)-0.5% sodium dodecyl sulfate at 60°C after hybridization, then incubated in 1:4000 diluted streptavidin-peroxidase (“HotStar, UK”) for 45–60 min at 42°. The membrane was then washed twice for 10 min in 2X SSPE-0.5% sodium dodecyl sulfate at 42°C and rinsed for 5 min in 2XSSPE at room temperature. The detection of hybridized DNA was done using the enhanced chemiluminescence method (“Amersham Biosciences, UK”) and by exposing it to X-ray film (“Hyperfilm ECL, Amersham Biosciences, UK”), following the manufacturer’s instruction. The presence or absence of spacers was visualized on X-ray film through the depiction of black and white squares, respectively. Reference strains, *M. bovis* BCG and MTB H37Rv used as positive controls, while sterile Qiagen water (“Qiagen Company, Germany”) was utilized as negative control.

### Statistical analysis

All the laboratory test data were first documented in a prepared “Microsoft Excel spreadsheet,” and checked if errors existed during recording (cleared), properly coded, then entered into STATA 15 (“Stata Corp, College Station, TX, USA”) for analysis. Descriptive statistical analysis, frequency, and percentage were used to summarize the key findings, and the Chi-square test’s *p*-value was reported. Findings were presented in tables and figures. The “shared international spoligotypes (SIT) number and the corresponding lineages/sublineages were obtained using the open-source spoligotype database (SpolDB4)^1^” (38). MTB Strains that exhibited similarity to a pre-existing spoligotype pattern in the database were assigned an SIT number, while TB strains without SIT numbers were categorized as “new.” MTB trains with the same spoligotype patterns was called a “cluster,” while a single pattern was defined as “unique” to this study.

### Research ethics

The study received ethical clearance from the Human Research Ethics Committee of the University of Pretoria, Faculty of Health Sciences, South Africa (Ref: No. 600/2018) and the National Research Ethics Review Committee, Ethiopia (Ref: No. SHE/SM/14.4/708/2019). Furthermore, a written official permission letter was obtained from the Ethiopian Orthodox Tewaheido Church (Ref: No.2478/6275/2011). A verbal and/or written consent declaration with full details about the study was given to participants and signed. Participants were assured that their data would remain confidential by using the non-personal identifier. Attendees who tested TB positive were linked with nearby healthcare facilities to commence the appropriate treatment and further management.

## Results

### Characteristics of study subjects

Five hundred and sixty PTB suspected cases participated in the study. Of these, 122 participants were bacteriologically confirmed (LJ culture-positive) (Supplementary Tables S1, S2). The median age of PTB suspected participants was 35 years, and male individuals constituted the majority (*n* = 308, 55.0%). One hundred twelve (20.0%), and 191 (31.1%) were previously treated and had a contact history with active TB patients, respectively (Supplementary Table S2). Of 122 bacteriologically confirmed TB cases, the majority (*n* = 33, 34.0%) and (*n* = 28, 26.7%) of the attendees were from the North Shewa and South Gondar zones, respectively. Over one-third (37.5 and 40.8%) of participants were previously treated and had contact history with active TB patients, respectively (Supplementary Tables S2, S3).

### Drug susceptibility testing

In this study, we have done gDST using “MTBDR*plus* VER2.0” on 122TB isolates. Of which 20 (16.4%%), and 15 (12.3%) were resistant to INH and RIF, respectively. Of those resistant to INH, 15 (75%) were also resistant to RIF and 5 (25%) were INH-monoresistant, while 1 (5%) isolate was INH-hetero-resistant. Three-fourths of drug-resistant isolates (*n* = 15, 75%) were MDR-TB (both RIF and INH resistant). The majority (*n* = 12, 16.0%) of them were identified from participants aged between 18 and 33 years, and male accounts (*n* = 9, 13.4%). Furthermore, 8 (36.4%) of those isolates identified as MDR-TB were detected from the South Wollo zone study site (Table 1).

**Table 1:** Proportions of anti-TB drug resistance with different variables (*n* = 122).

Variables			Anti-TB drug resistance					
		# of LJ-positive cases (n)	INH, n (%)	Chi-square (*p*-value)	RIF/MDR*, n (%)	Chi-square (*p*-value)	FLQs, n (%)	Chi-square (*p*-value)
Sex	Male	67	14 (20.9)	0.138	9 (13.4)	0.673	2 (3.0)	0.494
	Female	55	6 (10.9)		6 (10.9)		3 (5.5)	
Age group (years)	18–33	75	17 (22.7)	0.016	12 (16.0)	0.052	5 (6.7)	0.195
	34–49	31	0 (0.0)		0 (0.0)		0 (0.0)	
	≥50	16	3 (18.8)		3 (18.8)		0 (0.0)	
Residence	Urban	46	10 (21.7)	0.215	7 (15.2)	0.444	2 (4.3)	0.914
	Rural	76	10 (13.2)		8 (10.5)		3 (3.9)	
Marital status	Married	85	11 (12.9)	0.118	7 (8.2)	0.038	2 (2.4)	0.141
	Single*	37	9 (24.3)		8 (21.6)		3 (8.1)	
Educational status	Cannot read & write	59	6 (10.2)	0.132	4 (6.8)	0.080	0 (0.0)	0.010
	Primary school	30	8 (26.7)		7 (23.3)		4 (13.3)	
	Secondary school & above	33	6 (18.2)		4 (12.1)		1 (3.0)	
Family size	1–5	57	13 (22.8)	0.073	9 (15.8)	0.271	3 (5.3)	0.543
	>5	65	7 (10.8)		6 (9.2)		2 (3.1)	
Study zone	North Wello	22	1 (4.5)	0.005	1 (4.5)	0.005	0 (0.0)	<0.001
	South Wello	22	9 (40.9)		8 (36.4)		5 (22.7)	
	North Shewa	33	3 (9.1)		3 (9.1)		0 (0.0)	
	South Gondar	28	6 (21.4)		2 (7.1)		0 (0.0)	
	Central Gondar & others**	17	1 (5.9)		1 (5.9)		0 (0.0)	
Types of PTB cases	Previously treated	42	3 (7.1)	0.046	3 (7.1)	0.209	1 (2.4)	0.511
	Newly diagnosed	80	17 (21.3)		12 (15.0)		4 (5.0)	
Contact history with active TB patients	Yes	78	12 (15.4)	0.689	9 (11.5)	0.735	2 (2.6)	0.255
	No	44	8 (18.2)		6 (13.6)		3 (6.8)	

*Single, divorced & widowed; **Others: Awi zone, West Gojjam, East Gojjam, and Wag-Hamra. FLQs, Fluoroquinolones; INH, Isoniazid; LJ, Lowenstein-Jensen; MDR*, Multidrug-resistant (resistant to both RIF and INH); PTB, pulmonary tuberculosis; RIF, Rifampicin; TB, tuberculosis.

The MTBDR*sl* VER2.0 was done on 20 any INH-resistant (mono-hetero-multi/−) isolates and 15 RIF-resistant (mono-hetero-multi/−) MTB isolates. All of the isolates yielded interpretable results and were included in the study. Further resistance to FLQs was detected only in MDR-TB isolates. Accordingly, 25% (5/20) were identified as FLQs-resistant. Interestingly, all five (6.7%) FLQ-resistant isolates were detected from attendees aged between 18 and 33 years and from the South Wollo zone study area (Table 1).

### Frequency of gene mutations conferring resistance to INH, RIF, and FLQs

#### Mutations in the RIF-resistance determining region (*rpoB*)

In RIF-resistant isolates, the gene mutations in the *rpo*B were most frequent at codon Ser**531**Lue (80%); the next mutation was seen in His**526**Tyr (6.7%). The remaining two RIF-resistant isolates (13.3%) had shown a mutation or missing wild-type band (probe absent), but no corresponding MUT band, and were classified as “unknown” mutations. Those were *rpo*BWT6 and *rpo*BWT7 (6.7% each). This could be due to the gene mutations occurring outside the analyzed codon regions which LPAs cannot detect (Table 2).

**Table 2:** Gene mutations conferring resistance to RIF and INH.

Drug	Gene	Failing wild type band	Developing mutation band	Mutation	RR (*N* = 15) [n (%)]	MDR/RR (*N* = 15) [n (%)]
Rifampicin	*rpo*B	*rpo*BWT1	–	**–**	–	–
		*rpo*BWT2	–	**–**	–	–
		*rpo*BWT3	*rpo*BMUT1	D516V	–	–
		*rpo*BWT4	–	**–**	–	–
		*rpo*BWT5	–	–	–	–
		*rpo*BWT6	WT6 (absent)	Unknown	1 (6.7)	1 (6.7)
		*rpo*BWT7	WT7 (absent)	Unknown	1 (6.7)	1 (6.7)
		*rpo*BWT7	*rpo*BMUT2A	H526Y	1 (6.7)	1 (6.7)
			rpoBMUT2B	H526D	–	–
		*rpo*BWT8	*rpo*BMUT3	S531L	12 (80)	12 (80)
					MDR (Total, *N* = 15)	INH-MR (Total, *N* = 5)
					n (%)	n (%)
Isoniazid	*katG*	*kat*G WT	*kat*G MUT1	S315T1	15 (100)	5 (100)
		*kat*G WT	*kat*G MUT2	S315T2	–	–
	*inh*A	*inh*A WT1	*inhA* MUT1	C15T	–	–
			*inhA* MUT2	A15G	–	–
		*inh*A WT2	*inhA* MUT3A	T8C	–	–
			*inhA* MUT3B	T8A	–	–

INH, Isoniazid; MDR, Multidrug-resistant; INH-MR, Isoniazid Mono-resistant; MUT, Mutant; RIF, Rifampicin; RR, Rifampicin resistance; WT, Wild-type.

### Gene mutations in the *katG* and *inhA*

Amongst INH-resistant strains (*n* = 20), mutations in the *kat*G (indicating high-level resistance) were most frequent at codon Ser315Thr1 (95%), while no *inh*A gene mutation was observed. From 20 isolates with INH resistance, 5 (25.0%) were detected as INH-hetero-resistance and had mutations at codon Ser315Thr1 (100%) (Table 2).

Of 20 any drug-resistant isolates, 15 (75%) were MDR-TB strains (both RIF and INH- resistant). Of the total 15 MDR-TB strains, mutation in the *rpo*B and *kat*G genes were most frequent at Ser531Leu (*n* = 12, 80%) and Ser315Thr1 (*n* = 15, 100%) (Table 2). The remaining 3 MDR-TB isolates had shown a gene mutation at *rpo*BWT6 (probe absent) and Ser315Thr1, *rpo*BWT7 (probe absent) and Ser315Thr1, and His**526**Tyr and Ser315Thr1 (6.7% each) (Table 2).

### Gene mutations in the FLQ-resistance-determining region

The MTBDR*sl* VER2.0 was performed on 20 any drug-resistant TB strains to further assess the second-line injectable drugs (SLIDs) resistance. Accordingly, five MDR-TB strains had shown FLQs resistance, which is defined as pre-XDR-TB. All 5 FLQs-resistant isolates had mutations only in the *gyr*A genes (at codon Asp94Ala). No mutations were detected at *gyr*B, *rrs*, and *eis* high-level and low-level drug-resistance conferring gene mutations to SLIDs (Table 3).

**Table 3:** Gene mutations conferring resistance to FLQs.

Drug/phenotypic resistance	Gene	Failing wild type band	Developing mutation band	Mutation	MDR (*N* = 15) [n (%)]
FLQs	*gyr*A	*gyr*AWT1	–	–	–
		*gyr*AWT2	*gyr*AMUT1	A90V	–
			*gyr*AMUT2	S91P	–
		*gyr*AWT3	*gyr*AMUT3A	D94A	5 (33.3%)
			*Gyr*AMUT3B	D94N	–
				D94Y	–
			*gyr*AMUT3C	D94G	–
			*gyr*AMUT3D	D94H	–
	*gyr*B	*gyr*BWT	*gyr*BMUT1	N538D	–
			*gyr*BMUT2	E540V	–
KAN/AMK/CAP	*rrs*	*rrs*WT1	*rrs*MUT1	A1401G	–
KAN/CAP/VIO				C1402T	–
KAN/AMK/CAP/VIO		*rrs*WT2	*rrs*MUT2	G1484T	–
Low-level KAN	*eis*	*eis*WT1	–	G−37T	–
		*eis*WT2	*eis*MUT1	C−14T	–
			–	C−12T	–
			–	G−10A	–
		*eis*WT3	–	C−2A	–

AMK, Amikacin; CAP, Capreomycin; FLQs, Fluoroquinolones; KAM, Kanamycin; MUT, Mutant; MDR, Multidrug-resistant; VIO, Viomycin; WT, Wild-type.

### *Mycobacterium tuberculosis* genotypes and profiles of genetic changes conferring resistance to anti-TB drugs

To further analyze the genotype and clustering of 20 drug-resistant MTB strains, we performed a genotyping technique using the established Spoligotyping method, following the manufacturer’s protocol. All 20 drug-resistant MTB strains were successfully genotyped and have interpretable spoligo-patterns. The majority (*n* = 11, 55%) drug-resistant strains were found to be SIT149/T3-ETH sublineages followed by SIT21/CAS1-Kili (*n* = 6, 30%). The remaining isolates were CAS1-Delhi, CAS1-family, and SIT54/MANU2. Of the total 11 SIT149//T3-ETH sublineages, 4 strains were detected from the South Gondar zone study site, while 2 and 3 isolates were detected from the South Wello and North Shewa zones, respectively. Also, from six SIT21/CAS1-Kili sublineages, four isolates were identified from the South Wello zone study site, while the remaining two isolates were from the North Shewa and North Wello zone study area (Table 4).

**Table 4:** Drug-resistant *M. tuberculosis* lineages and profiles of the gene mutations conferring resistance to INH, RIF, MDR/RR, and FLQs.

Sample ID	Isolation zone	Spoligotypes descriptions (Binary format)	SIT No	Lineage/clades	Any INH-r (*n* = 20)	Any RR (*n* = 15)				MDR/RR (*n* = 15)				FLQ-r (*n* = 5)
					S315T1	S531L	H526Y	WT6 (absent)	WT7 (absent)	S531L & S315T1	*rpo*BWT7 & S315T1	H**526**Y & S**315**T1	*rpo*BWT6 & S**315**T1	D94A, S531L & S315T1
NW91	NW	■■■□□□□■■□■■■■■■■■■□□□□□□□□□□□□□□□□■■■■■■■■	21	CAS1-Kili			–	–	–		–	–	–	–
SW02	SW	■■■□□□□■■□■■■■■■■■■□□□□□□□□□□□□□□□□■■■■■■■■	21	CAS1-Kili			–	–	–		–	–	–	
SW86	SW	■■■□□□□■■□■■■■■■■■■□□□□□□□□□□□□□□□□■■■■■■■■	21	CAS1-Kili			–	–	–		–	–	–	
SW91	SW	■■■□□□□■■□■■■■■■■■■□□□□□□□□□□□□□□□□■■■■■■■■	21	CAS1-Kili			–	–	–		–	–	–	
SW100	SW	■■■□□□□■■□■■■■■■■■■□□□□□□□□□□□□□□□□■■■■■■■■	21	CAS1-Kili			–	–	–		–	–	–	
SW43	SW	■■■■■■■■■□□□□□□□□□□■■■■■■■■■■■■■□□□□■■■■■■■	149	T3-ETH			–	–	–		–	–	–	–
SW94	SW	■■■■■■■■■□□□□□□□□□□■■■■■■■■■■■■■□□□□■■■■■■■	149	T3-ETH			–	–	–		–	–	–	–
SW97	SW	■■■■■■■■■□□□□□□□□□□■■■■■■■■■■■■■□□□□■■■■■■■	149	T3-ETH			–	–	–		–	–	–	–
SW67	SW	■■■□□□□■■■■■■■■■■■■■■■□□□□□□□□□□□□■■□□■■■■■	25	CAS1-Delhi*		–	–	–	–	–	–	–	–	–
SW69	SW	■■■□□□□■■□■■■□■■■■■□□□□□□□□□□□□□□□□■■■■■■■■	NA	CAS family			–	–	–					
NS48	NS	■■■■■■■■■□□□□□□□□□□■■■■■■■■■■■■■□□□□■■■■■■■	149	T3-ETH**		–	–	–		–		–	–	–
NS73	NS	■■■■■■■■■□□□□□□□□□□■■■■■■■■■■■■■□□□□■■■■■■■	149	T3-ETH		–		–	–	–	–		–	–
NS63	NS	■■■□□□□■■□■■■■■■■■■□□□□□□□□□□□□□□□□■■■■■■■■	21	CAS1-Kili			–	–	–		–	–	–	–
SG16	SG	■■■■■■■■■□□□□□□□□□□■■■■■■■■■■■■■□□□□■■■■■■■	149	T3-ETH			–	–	–		–	–	–	–
SG35	SG	■■■■■■■■■□□□□□□□□□□■■■■■■■■■■■■■□□□□■■■■■■■	149	T3-ETH			–	–	–		–	–	–	–
SG97	SG	■■■■■■■■■□□□□□□□□□□■■■■■■■■■■■■■□□□□■■■■■■■	149	T3-ETH*		–	–	–	–	–	–	–	–	–
SG98	SG	■■■■■■■■■□□□□□□□□□□■■■■■■■■■■■■■□□□□■■■■■■■	149	T3-ETH*		–	–	–	–	–	–	–	–	–
SG99	SG	■■■■■■■■■□□□□□□□□□□■■■■■■■■■■■■■□□□□■■■■■■■	149	T3-ETH*		–	–	–	–	–	–	–	–	–
SG100	SG	■■■■■■■■■□□□□□□□□□□■■■■■■■■■■■■■□□□□■■■■■■■	149	T3-ETH*		–	–	–	–	–	–	–	–	–
WG31	WG	■■■■■■■■■■■■■■■■■■■■■■■■■■■■■■■■□□■■■■■■■■■	54	MANU2		–	–		–	–	–	–		–

**Isoniazid hetero-resistant; *Isoniazid-mono-resistant; The presence of gene mutations conferring drug-resistance in each *M. tuberculosis* lineages/sublineages is highlighted in green color. INH-r, Isoniazid resistant; MDR, Multidrug resistance; MUT, Mutant; NA, Not assigned; NS, North Showa; NW, North Wello; SG, South Gondar; SIT, Spoligo-international types; SW, South Wello; RR, Rifampicin resistance; FLQ-r, Fluoroquinolone resistance; WT, Wild-type; WG, West Gojjam.

Furthermore, of 11 SIT149/T3-ETH sublineages, 7 strains were MDR-TB, while four were INH-mono-resistant. Amongst 6 CAS1-Kili/SIT21 and one CAS1-family sublineages, all were MDR-TB, while four of these were FLQs-resistant strains. The remaining two sublineages, SIT54/MANU2 and SIT25/CAS1-Delhi were MDR and INH-mono-resistant, respectively (Table 4).

Amongst 7 MDR SIT149 genotypes, five had genetic changes in *rpo*B and *kat*G genes (at codon Ser531Leu and Ser315Thr1), while one isolate had a gene mutation at codon His**526**Tyr and Ser**315**Thr, and four isolates were INH-mono-resistant with a gene mutation in *kat*G (at codon Ser315Thr1). All the six MDR SIT21/CAS1-Kili and CAS1-family spoligotypes had drug resistance-conferring gene mutations in *rpo*B and *kat*G genes (at codon Ser531Leu and Ser315Thr1), of these, four isolates were FLQs-resistant with a drug-resistance conferring gene mutations in *rpo*B, *kat*G, and *gyr*A genes (at codon Ser531Leu, Ser315Thr1, D94A, respectively) (Table 4).

## Discussion

Early diagnosis and effective anti-mycobacterial drugs targeting the infecting MTB isolate are critical for improving patient management, increasing cure rates, and limiting further transmission of the disease (1, 3). Moreover, it is crucial to identify the key genetic changes responsible for anti-TB drug resistance and the associated drug-resistant MTB genotypes to handle patients with DR-TB effectively (2, 3, 39). Nowadays, the innovation and improvements of gDST methods capable of evaluating genes harboring antimicrobial drug resistance mutations have substantially improved the detection and management of drug-resistant TB (14, 15). In addition to XpertMTB/RIF assay, LPAs are commonly used in developing countries including Ethiopia, and come at the forefront in performing gDST and evaluating the occurrence of specific gene mutations resulting in drug resistance to some key anti-mycobacterial medicines used in the treatment of drug-susceptible and resistant TB strains (14–16, 40). Thus, the widespread gDST service is very important for tailoring TB patient treatment and hindering the transmission of the disease.

The most common gene mutations linked with RIF resistance occur in the 81 bp RIF-resistance determining region (RRDR) (codons 507–533) of the *rpo*B gene (95%), which encodes the “RNA polymerase beta-subunit” (2, 41). Those gene mutations alter the binding site of RIF, preventing its inhibitory action on RNA synthesis (13, 41, 42). Earlier studies have shown that specific mutations, at codons S531L, H526Y, and D516V are frequently observed in RIF-resistant MTB (13, 21, 43–45). In this study, among RIF-resistant TB isolates, the most frequent mutation (80%) associated with RIF resistance was at codon S531L, followed by His526Tyr (6.7%). The gene mutation at codon S531L was noted as the dominant mutation of the *rpo*B gene responsible for RIF resistance in several previous studies done in different parts of Ethiopia; which includes the Amhara region (73%) (46), Tigray region (70%) (44), Somali region (80%) (47), Addis Ababa (81.3%) (48), Southwest Ethiopia (82.4%) (49), a meta-analysis report, Ethiopia (74.2%) (21), and a recent multicenter study report, Ethiopia (59.1%) (45). In line with our result, a higher frequency of mutation at codon S531L was reported in other high TB burden countries like China (58%) (50), India (62%) (51), Pakistan (64%) (52), Sudan (64%) (53), and Iran (66%) (54). The other mutation in our study, His526Tyr was previously reported in Ethiopia, which ranged from 6.6–17.2% (21, 44, 45, 55), Sudan (12.8%) (53), India (11.1%) (51), and China (8.9%) (50). However, a similar or higher proportion to our results was noted in Sudan (12.8%) (53), India (11.1%) (51), China (8.9%) (50), South Africa, at amino acid position 526 (27.9%) (36). In this study, two RIF-resistant isolates (*rpo*BWT6 and *rpo*BWT7) had shown a mutation or missing WT band, but no corresponding MUT band, and were classified as unknown genetic changes. This proportion of isolates with unknown mutations is similar to other studies from a recent multicenter study, Ethiopia (13.6%) (45), Addis Ababa, Ethiopia (15.8%) (55), Southern Ethiopia (14.7%) (56), and elsewhere, a large, multisite diagnostic study (13%) (57). This could be due to the mutations occurring outside the analyzed codon regions (drug-resistance determining regions) which LPAs unable to evaluate/detect (58).

Isoniazid resistance is mostly due to the genetic change in the *kat*G gene (50–95%) and the *inh*A promoter region (20–35%) (2, 13, 41, 51), depending on geographical distributions (59). The gene mutations in the *kat*G gene inhibit INH activation, while the gene mutations in the *inh*A promoter region result in overexpression of the *inh*A gene, which encodes the INH target enzyme (13, 41). Prominent genetic changes associated with INH resistance include *kat*G Ser315Thr1/2, and *inh*A -15C/T (44, 60). Similarly, in the current study, amongst INH-resistant isolates, mutations in the *kat*G was most frequent at codon Ser315Thr1 (95%), while no *inh*A gene mutation was observed. In agreement with our findings, a recent multicenter study in Ethiopia reported a prevalence of 91.8% for *kat*G/Ser315Thr1 mutation (45), and a meta-analysis study that examined INH conferring gene mutations noted a prevalence of 95.8% for *kat*G315 mutation (21). The *kat*G/Ser315Thr1 gene mutation, causing high-level INH resistance was predominant (95%) in the current study and other earlier studies in different parts of Ethiopia (21, 45–48, 55, 56, 61). “Mutations in the *inh*A promoter region which are associated with low-level INH resistance are usually less frequent when compared with *kat*G mutations” (41). The *inh*A gene mutation is not observed in our study; as well as other studies in Ethiopia also reported no or low proportion of mutation in the *inh*A promotor region (21, 46, 48, 55, 61).

The indiscriminate use of FLQs in many countries including Ethiopia for various common infectious disease treatments, which might result in the development of antimicrobial resistance against these key antibiotics (62–64). In FLQ-resistant MTB isolates, mutations in the *gyr*A and *gyr*B genes, which encode the DNA gyrase enzyme, are commonly observed (20, 42, 65). These mutations alter the binding site of FLQs, reducing their inhibitory effect on DNA replication. Specific mutations, at codons, such as D94G, A90V, and S91P of *gyr*A (quinoline resistance-determining region, QRDR) have been frequently reported in FLQ-resistant TB (11, 45, 65). In the current study, amongst 15 MDR-TB isolates, 5 (33.3%) were FLQs-resistant, and all isolates had *gyr*A gene mutations at codon Asp94Ala(D/A). Supporting our finding, a recent multisite study conducted in Ethiopia reported that *gyr*A/Asp94Ala gene mutation (14.3%) was detected in FLQs-resistant TB isolates in Ethiopia (45). However, in contrast to our observation, studies in Ethiopia reported that *gyr*A/D94G gene mutations were predominant in FLQs-resistant TB isolates (44, 66, 67), while few other studies indicated that *gyr*A/A90V gene mutations are prevalent and responsible for FLQs-resistance in Ethiopia (45, 62). Several previous studies have indicated that *gyr*A/D94G gene mutation is predominant across the corners of the globe (68–72). However, further molecular study data is necessary to fully understand the spectrum of gene mutations that confer resistance to FLQs (gyrA/B gene mutations) on MTB in Ethiopia. In our study, *gyr*B gene mutation was not observed, which is in concordance with previous studies in Ethiopia (44, 45, 62).

The observed association between MTB genotypes and drug resistance in Ethiopia highlights the importance of understanding the genetic diversity of the pathogen in combating drug-resistant TB (63, 73, 74). The diversity of MTB genotypes circulating in the country, coupled with specific mutations conferring drug resistance, underscores the need for a comprehensive approach to TB control (73, 75). A lack of comprehensive data on the molecular epidemiology of DR-TB strains in Ethiopia further complicates the implementation of molecular diagnostics. Yet, in Ethiopia, the link between resistance to anti-TB drugs and MTB genotypes is a complex and multifactorial phenomenon (75). Thus, characterizing DR-TB genotypes and understanding the prevalence and distribution of resistance-associated gene mutations for key anti-TB drugs is essential for guiding treatment strategies and monitoring the emergence of new resistance patterns in the country (21).

In the current study, 20 drug-resistant MTB isolates were successfully Spoligotyped, in which the majority (55%) were SIT149/T3-ETH Sublineages followed by SIT21/CAS1-Kili (30%). Furthermore, from 11 SIT149/T3-ETH sublineages, 7 were MDR-TB, while four were INH-hetero-resistant. Supporting our findings, the high clustering rate and predominance of drug-resistant SIT149/T3-ETH sublineages were reported in Ethiopia (56, 63, 76, 77). In agreement with our findings, earlier studies in Ethiopia reported that SIT149/T3-ETH sublineages were predominantly linked with drug resistance, particularly MDR/XDR-TB (63, 73–75, 78). However, in contrast to our findings, a recent study in Ethiopia noted that “TUR-genotype (54%) was predominant in MDR-TB strains” (79). This could be the fact that the T3-ETH/SIT149 Sublineages is an Ethiopian-specific genotype that predominantly circulates in the highlands of the country and plays an important role in TB disease transmission (73). Thus, a comprehensive study using improved genotyping techniques such as sequencing methods with high discriminatory power is warranted to tailor the clustering of these sublineages and their association with drug resistance. However, the predominance and its association with drug-resistance, SIT149/T3-ETH sublineages could indicate that these strains are becoming more important in TB disease transmission and developing drug resistance in Ethiopia. On the other hand, high proportions of holy water site attendees infected with clustered TB strains, suggest probable recent transmission of MDR-TB in the study area. Furthermore, SIT21/CAS1-Kili sublineages were the second most prevalent in the current study, in which among 6 CAS1-Kili and one CAS-family sublineages, all strains were MDR-TB, and four of these were FLQs-resistant. In line with our observation, a few studies conducted in Ethiopia (63, 80), and elsewhere, in Zambia (81), reported that SIT21 sublineages were frequently detected in MDR/XDR-TB isolates. The emergence of drug resistance in the Ethiopian strains, CAS1-Kili/SIT21 and T3-ETH/SIT149, could potentially be attributed to local factors including delayed diagnosis, inadequate compliance, insufficient contact investigations, or other unidentified factors TB prevention and care system.

### Limitations

However, this study has limitations. The number of TB isolates included in our study is relatively very small, and study population selection bias might impede generalizing the findings. Since LPAs can only detect the presence/absence of specific gene mutations responsible for drug resistance for key anti-TB drugs, it will not be enough to describe the spectrum of gene mutations in the country or study region. We used only the Spoligotyping technique to characterize the genetic diversity of drug-resistant MTB isolates, which may have resulted in low discriminatory capacity and hampered identification of transmission chains.

## Conclusion

The finding of our study revealed that canonical drug resistance-conferring gene mutations at *rpo*B/S531L, *kat*G/S315T1, and *gyr*A/D94G were the most frequent in RIF, INH, and FLQs-resistant isolates, respectively. In this study, higher clustering rates of drug-resistant MTB lineages were observed. Furthermore, two sublineages, SIT149/T3-ETH and CAS1-Kili showed a higher proportion of drug resistance, particularly MDR/pre-XDR-TB. However, to comprehend better the association of SIT149/T3-ETH and SIT21/CAS1-Kili sublineages with drug resistance in Ethiopia, improved genotyping techniques with high discriminatory power such as sequencing methods and a large number of molecular data is warranted to further elucidate such genotypes and mutations and predict drug-resistance. TB screening and surveillance for drug resistance among key populations are essential to effectively control the disease in the country.

## Data availability statement

The original contributions presented in the study are included in the article/Supplementary material, further inquiries can be directed to the corresponding author.

## Ethics statement

The studies involving humans were approved by the Human Research Ethics Committee of the University of Pretoria, Faculty of Health Sciences, South Africa (Ref: No.600/2018). The studies were conducted in accordance with the local legislation and institutional requirements. The participants provided their written informed consent to participate in this study.

## Author contributions

MAR: Writing – review & editing, Writing – original draft, Visualization, Validation, Software, Resources, Project administration, Methodology, Investigation, Funding acquisition, Formal analysis, Data curation, Conceptualization. NEM: Writing – review & editing, Writing – original draft, Visualization, Validation, Supervision, Software, Resources, Investigation, Funding acquisition, Formal analysis, Data curation, Conceptualization. PBF: Writing – review & editing, Visualization, Validation, Supervision, Software, Resources, Project administration, Methodology, Investigation, Funding acquisition, Formal analysis, Data curation, Conceptualization.

## Footnotes

^1^ https://www.pasteur-guadeloupe.fr:8081/SITVITDemo/
