# Supplementary material for: Patterns and profiles of drug resistance-conferring mutations in Mycobacterium tuberculosis genotypes isolated from tuberculosis-suspected attendees of spiritual holy water sites in Northwest Ethiopia
Source: Front Public Health. 2024 Mar 19;12:1356826. doi: 10.3389/fpubh.2024.1356826 (PMC10985251; doi:10.3389/fpubh.2024.1356826)
Supplement: Supplementary file 1 [file Data_Sheet_1.pdf]

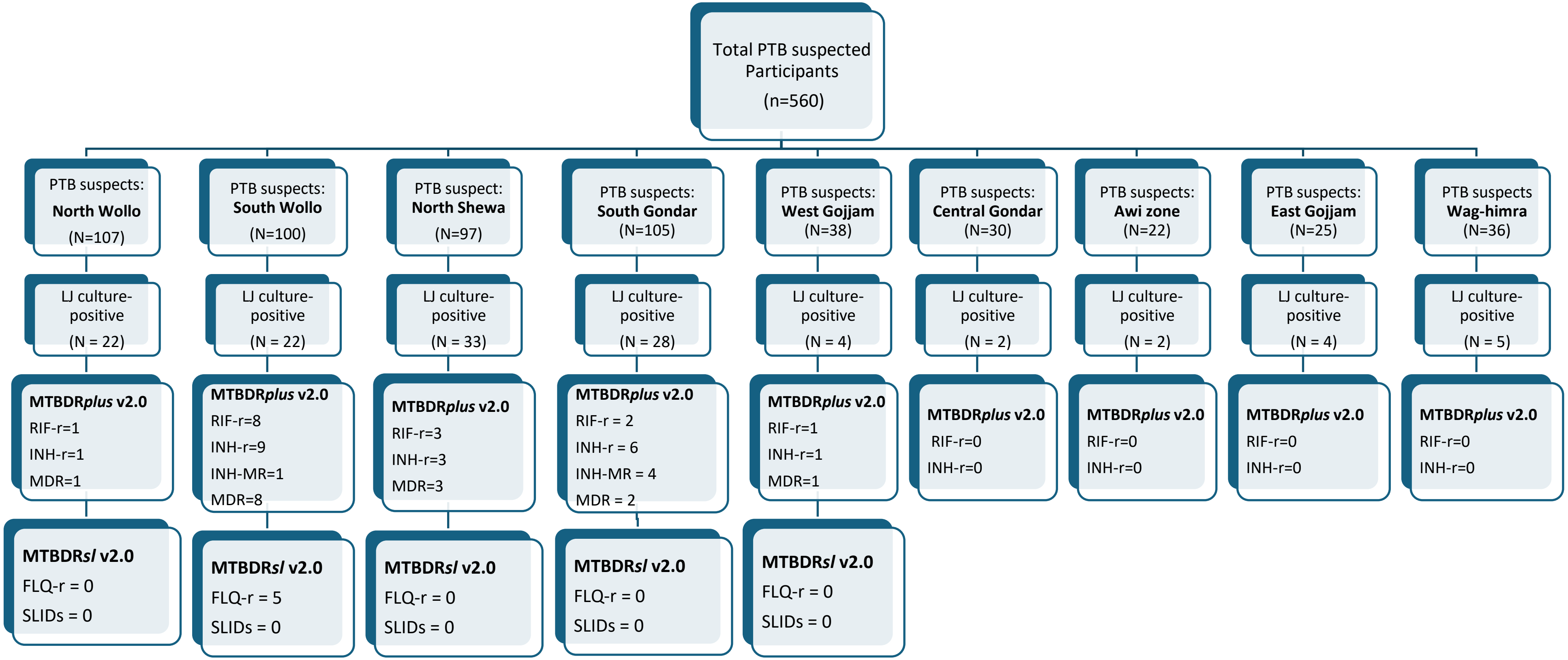

Figure S1: *Study population, bacteriologically confirmed TB cases from each study zone, and detection of first- and second-line anti-TB drug resistance.* ***Abbreviations***: ***FLQ-r***: *Fluoroquinolone-resistance;* ***INH-MR***: *Isoniazid-monoresistance;* ***INH-r***: *Isoniazid-resistance;* ***LJ***: *Lowenstein-Jensen;* ***MDR***: *Multidrug-resistance (i.e., RIF-r + INH-r);* ***PTB***: *Pulmonary tuberculosis;* ***RIF-r***: *Rifampicin-resistance;* ***SLIDs***: *Second-line injectable drugs; MTBDRplus v2.0 (GenoType®MTBDRplus VER2.0); MTBDRsl v2.0 (GenoType®MTBDRsl VER2.0).*
