# Supplementary material for: Patterns and profiles of drug resistance-conferring mutations in Mycobacterium tuberculosis genotypes isolated from tuberculosis-suspected attendees of spiritual holy water sites in Northwest Ethiopia
Source: Front Public Health. 2024 Mar 19;12:1356826. doi: 10.3389/fpubh.2024.1356826 (PMC10985251; doi:10.3389/fpubh.2024.1356826)
Supplement: Supplementary file 2 [file Table_1.docx]

**Table S1**: The study area (administrative zones), selected spiritual holy water sites, total number of attendees screened for PTB suggestive symptoms, enrolled participants in each study site, and LJ culture test results, 2019-2020.

| S. No | Study area (zones) | Holy Water Site (HWS) | # of attendees screened for PTB symptoms | # of PTB suspected case | Bacteriologically confirmed PTB cases (LJ positive) | Culture negative case (LJ negative) |
| --- | --- | --- | --- | --- | --- | --- |
| 1 | North-Wello | *Urael* | 1458 | 107 | 22 | 85 |
| 2 | South-Wello | *Amanual* | 1601 | 100 | 22 | 78 |
| 3 | North-Shewa | *Tsadkane Mariam* | 3221 | 97 | 33 | 64 |
| 4 | South-Gondar | *Fogera -Arsema* | 1201 | 105 | 28 | 77 |
| 5 | Central-Gondar | *Teklehymanot* | 820 | 30 | 2 | 28 |
| 6 | Awi zone | *Ashewa Medihanealem* | 530 | 22 | 2 | 20 |
| 7 | West-Gojam | *Zera-biruk* | 471 | 38 | 4 | 34 |
| 8 | Wag-Hamra | *Gihorgis* | 543 | 36 | 5 | 31 |
| 9 | East-Gojam | *Washa-Giyorgis* | 468 | 25 | 4 | 21 |
|  | **Total** | | **10, 313** | **560** | **122** | **438** |
| *Note: PTB: Pulmonary tuberculosis; LJ: Lowenstein-Jensen; HWS: Holy water sites.* | | | | | | |

**Table S2**: Proportion of bacteriologically confirmed TB cases with socio-demographic characteristics of participants (n = 560).

| Variables | | |  | Bacteriologically confirmed TB cases | | Chi-square  (*p*-value) |  |
| --- | --- | --- | --- | --- | --- | --- | --- |
|  |  |  | **# of TB suspected cases** | **Positive,**  **n (%)** | **Negative,**  **n (%)** |  |  |
| Sex | Male | | 308 | 67 (21.8) | 241(78.2) | 0.984 |  |
|  | Female | | 252 | 55 (21.8) | 197 (78.2) |  |  |
| Age group (year) | 18-33 | | 263 | 75 (28.5) | 188 (71.5) | 0.001 |  |
|  | 34-49 | | 208 | 31 (14.9) | 177 (85.1) |  |  |
|  | ≥ 50 | | 89 | 16 (18.0) | 73 (82.0) |  |  |
| Residence | Urban | | 258 | 46 (17.8) | 212 (82.2) | 0.036 |  |
|  | Rural | | 302 | 76 (25.2) | 226 (74.8) |  |  |
| Marital status | Married | | 356 | 85 (23.9) | 271 (76.1) | 0.113 |  |
|  | Single* | | 204 | 37 (18.1) | 167 (81.9) |  |  |
| Educational status | Can't read & write | | 256 | 59 (23.0) | 197 (77.0) | 0.405 |  |
|  | Primary school | | 165 | 30 (18.2) | 135 (81.8) |  |  |
|  | Secondary school & above | | 139 | 33 (23.7) | 106 (76.3) |  |  |
| Household size | 1-5 | | 294 | 57 (19.4) | 237 (80.6) | 0.148 |  |
|  | > 5 | | 266 | 65 (24.4) | 201 (75.6) |  |  |
| Occupation | Farmer | | 235 | 45 (19.1) | 190 (80.9) | 0.486 |  |
|  | Employed | | 24 | 6 (25.0) | 18 (75.0) |  |  |
|  | Unemployed | | 117 | 23 (19.7) | 94 (80.3) |  |  |
|  | Housewife | | 94 | 24 (25.5) | 70 (74.5) |  |  |
|  | Students & others ****** | | 90 | 24 (26.7) | 66 (73.3) |  |  |
| Study zone | North Wello | | 107 | 22 (20.6) | 85 (79.4) | 0.001 |  |
|  | South Wello | | 100 | 22 (22.0) | 78 (78.0) |  |  |
|  | North Shewa | | 97 | 33 (34.0) | 64 (66.0) |  |  |
|  | South Gondar | | 105 | 28 (26.7) | 77 (25.7) |  |  |
|  | Central Gondar & others*** | | 151 | 17 (11.3) | 134 (88.7) |  |  |
| Types of TB cases | Previously treated | | 112 | 42 (37.5) | 70(62.5) | < 0.001 |  |
|  | Newly diagnosed | | 448 | 80(17.9) | 368(82.1) |  |  |
| Contact history with active TB patients | Yes | | 191 | 78 (40.8) | 113 (59.2) | < 0.001 |  |
|  | No | | 369 | 44 (11.9) | 325 (88.1) |  |  |
| *Note: *Single, divorced & widowed; **religious leaders & deacons*; ****Others: Awi zone, West Gojjam, East Gojjam, and Wag-Hamra; TB: Tuberculosis* | | | | | | |  |
|  | |  | | | | |  |

**Table S3**: Sociodemographic characteristics of bacteriologically confirmed TB cases with any anti-TB drug resistance patterns (n = 122).

| Variables | |  | Any anti-TB drug resistance | | | |
| --- | --- | --- | --- | --- | --- | --- |
|  |  | **# of PTB-positive cases (n)** | **Yes, n (%)** | **No, n(%)** | **Chi-square**  **(*p*-value)** |  |
| Sex | Male | 67 | 14 (20.9) | 53 (79.1) | 0.138 |  |
|  | Female | 55 | 6 (10.9) | 49 (89.1) |  |  |
| Age group (years | 18-33 | 75 | 17 (22.7) | 58 (77.3) | 0.016 |  |
|  | 34-49 | 31 | 0 (0.0) | 31 (100.0) |  |  |
|  | ≥ 50 | 16 | 3 (18.8) | 13 (81.3) |  |  |
| Residence | Urban | 46 | 10 (21.7) | 36 (78.3) | 0.215 |  |
|  | Rural | 76 | 10 (13.2) | 66 (86.8) |  |  |
| Marital status | Married | 85 | 11 (12.9) | 74 (87.1) | 0.118 |  |
|  | Single* | 37 | 9 (24.3) | 28 (75.7) |  |  |
| Educational status | Can’t read & write | 59 | 6 (10.2) | 53 (89.8) | 0.132 |  |
|  | Primary school | 30 | 8 (26.7) | 22 (73.3) |  |  |
|  | Secondary school & above | 33 | 6 (18.2) | 27 (81.8) |  |  |
| Family size per household | 1-5 | 57 | 13 (22.8) | 44 (77.2) | 0.073 |  |
|  | > 5 | 65 | 7 (10.8) | 58 (89.2) |  |  |
| Study zone | North Wello | 22 | 1 (4.5) | 21 (95.5) | 0.005 |  |
|  | South Wello | 22 | 9 (40.9) | 13 (59.1) |  |  |
|  | North Shewa | 33 | 3 (9.1) | 30 (90.9) |  |  |
|  | South Gondar | 28 | 6 (21.4) | 22 (78.6) |  |  |
|  | Central Gondar & others** | 17 | 1 (5.9) | 16 (94.1) |  |  |
| Types of PTB cases | Previously treated | 42 | 3 (7.1) | 39 (92.9) | 0.046 |  |
|  | Newly diagnosed | 80 | 17 (21.3) | 63 (78.8) |  |  |
| Contact history with active TB patients | Yes | 78 | 12 (15.4) | 66 (84.6) | 0.689 |  |
|  | No | 44 | 8 (18.2) | 36 (81.8) |  |  |
| Note: **Single, divorced & widowed; **Others: Awi zone, West Gojjam, East Gojjam, and Wag-Hamra. Any anti-TB drug resistance (i.e., resistance to INH and/or RIF, Fluoroquinolones (FLQs)). Abbreviations: INH: Isoniazid; PTB: pulmonary tuberculosis; RIF: Rifampicin; TB: tuberculosis.* | | | | | | |
